# Examining dietitians' knowledge, skills and attitudes regarding working with older adults in residential aged care facilities and home care services: An integrative review

**DOI:** 10.1111/jhn.13073

**Published:** 2022-08-22

**Authors:** Karly Bartrim, Wendy Moyle, Roshan Rigby, Lauren Ball

**Affiliations:** ^1^ Menzies Health Institute Queensland Griffith University Southport QLD Australia; ^2^ School of Health Sciences and Social Work Griffith University Southport QLD Australia; ^3^ School of Nursing and Midwifery Griffith University Nathan QLD Australia

**Keywords:** aged, attitude, dietitians, home care services, nursing homes, residential facilities

## Abstract

**Background:**

The Australian 2021 Royal Commission identified that the dietetic workforce needs to grow in size and capacity to support nutrition care in older adults. However, little is known about dietitians' knowledge, skills and attitudes (KSA) regarding working with older adults in residential aged care facilities (RACFs) or their homes. This review describes dietitians' KSA regarding older adults in RACFs and home care services.

**Methods:**

A systematic literature search was conducted in August 2021 to identify studies examining any aspect of dietitians or student dietitians' KSA working in RACFs and home care services. No restrictions were applied to methodological design, language, location or publication year. Studies were assessed for quality using the Johanna Briggs Institute Quality Appraisal Tools. Study findings were analysed thematically using meta‐synthesis.

**Results:**

All 17 studies that met the inclusion criteria explored dietitians' attitudes towards their role, three studies examined perceived knowledge, although no studies objectively explored dietitians' skill levels. Five themes were developed inductively: (1) recognising their contribution as dietitians; (2) lacking clarity about the boundaries of their role; (3) all team members have a role to play in nutrition care; (4) assumptions and biases about working with older people; and (5) needing to build capacity in the workforce.

**Discussion:**

Dietitians have mixed attitudes about working in RACFs and home care services. Future directions include evaluating dietitians' role in RACFs, reviewing education and training and practical opportunities for student dietitians, and assessing the impact of more dietitian support on an older person's dietary intake and nutrition.

## Key points

- Dietitians have mixed attitudes about working in residential aged care facilities and home care services.
- There is an opportunity to further explore dietitians' knowledge and skills about working with older adults.

## Graphical abstract

Attitudes of dietitians working with older adults.

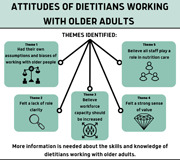

## INTRODUCTION

The ageing population continues to place demand for older adults requiring care in residential aged care facilities (RACFs) and in‐home health care.^1^, ^2^, ^3^, ^4^ In Australia, the 2021 Royal Commission into Quality and Safety in Aged Care recognised an urgent need to improve services that support Australians to live and age well.^2^ Recommendations from the Royal Commission included strengthening the workforce that cares for older adults, increasing employment of allied health professionals in aged care services and ensuring equity of care for older adults living at home.^2^

Dietitians are the only health professionals trained in providing nutrition care to prevent or reduce age‐related malnutrition and chronic disease.^5^ Through delivery of nutrition interventions, dietitians are able to improve the health and wellbeing of older adults.^6^, ^7^ Therefore, they are an important workforce for providing nutrition care in RACFs and in‐home services. In response to the Royal Commission, Dietitians Australia recommended that RACFs engage dietitians to provide at least 1 h per month of nutrition care per resident.^8^ If this recommendation is to be achieved, the dietetic workforce in aged care needs to increase substantially in size and capacity.^7^

Currently, little is known about dietitians' knowledge, skills and attitudes (KSA) regarding working with older adults in RACFs or their homes. A greater understanding of dietitians' KSA when working with older adults in RACFs or their homes can inform workforce strategies to support capacity‐building initiatives that produce quality care. Therefore, the present study aimed to synthesise evidence regarding dietitians' KSA working in RACFs and home care services.

## METHODS

An integrative review was conducted following the methodology of Whitemore and Knafl^9^ to examine dietitians working with older adults in RACFs and home care services. An integrative review was chosen as a result of the range of study designs, including qualitative and quantitative studies.^9^ The five‐stage methodology framework consists of (1) problem identification; (2) a comprehensive search of peer reviewed literature; (3) data evaluation such as study aim, methods, participants, setting, inclusion and exclusion criteria; (4) data analysis using meta‐synthesis; and (5) presentation to identify knowledge and evidence gaps to inform future research and practice.^9^ The framework enabled the results from various methodologies to be integrated, forming a synthesis of dietitians KSA with older people in these settings, which provides new knowledge to this area of dietetic practice.^9^ The Preferred Reporting Items for Systematic Reviews and Meta‐Analyses (PRISMA) guidelines were followed to report the findings.^10^

### Search strategy

A systematic literature search was conducted in August 2021, with an additional search conducted in January 2022 to capture any new articles. Online databases were searched to identify potentially relevant documents in Scopus, Web of Science, PubMed, CINAHL Complete (EBSCOhost) and Health and Medicine (ProQuest). All of the researchers developed the search strategy with guidance from an experienced health librarian. Literature was searched using the following terms (‘dietitian’ OR ‘dietician’ OR ‘nutritionist’) AND (‘nursing home’ OR ‘nursing facilit*’ OR ‘aged care’ OR ‘long term care’ OR ‘residential care’ OR ‘residential facilit*’ OR ‘old peoples home’ OR ‘care home’ OR ‘retirement home’ OR ‘retirement facilit*’ OR ‘home‐based’ OR ‘home visit’ OR ‘home care’ OR ‘home service’ OR ‘domiciliary service*’ OR ‘domiciliary care’ OR ‘community care’ OR ‘community service’ OR ‘outreach service’) AND (‘knowledge*’ OR ‘skill*’ OR ‘attitude*’). In applicable databases, the following MeSH terms were applied (‘Home Care Services’ OR ‘Nursing Homes’ OR ‘Home Nursing’ OR ‘Long‐Term Care’ OR ‘Community Health Services’ OR ‘Residential Facilities’).

### Study criteria

#### Inclusion criteria

Studies were eligible for inclusion if they examined any aspect of participants (dietitians, student dietitians or nutritionists) KSA regarding working in RACFs and home care services with older adults (> 65 years). RACFs encompassed nursing home facilities, long‐term care facilities, residential care or facility, ‘old people's homes’, care homes and retirement homes or retirement facilities, as well as care provided via home visits or domiciliary services to older (> 65 years) ambulatory patients living in the community. No restrictions were applied to methodological design, language, geographical location or year of publication. The review did not include rehabilitation or respite settings because they are often associated with a hospital service. Studies were excluded if data on dietitians could not be separated from other health professionals, such as nursing staff. Studies where the aged care or community dietitian data could not be isolated from dietitians working in other settings (e.g, hospital‐based dietitians) were also ineligible for inclusion.

#### Types of evidence

All articles were imported from the databases into Endnote (https://endnote.com) and duplicates were removed. The remaining articles were exported into Covidence (https://www.covidence.org), which is an online tool that streamlines parts of the review process). Within Covidence, study titles and abstracts were screened independently in duplicate. One researcher (KB) screened all titles and abstracts, and two other members of the research team (LB and RR) shared the screening of all titles and abstracts. The research team comprised three academic dietitians and an academic nurse. One dietitian has experience working in residential aged care and home care settings, and the registered nurse is gerontological trained with clinical experience in residential aged care. Full‐text articles were screened in duplicate independently against the selection criteria by KB and RR. Articles not in English were sent to bilingual colleagues for translation. Finally, the reference lists of all included studies were hand‐searched for any additional relevant studies.

#### Data charting process and items

Data from the identified studies were extracted within Covidence using a predesigned template. Data were extracted by one researcher (KB) and checked for accuracy by a second researcher (LB). Data included article details and characteristics (e.g., author, year, country of origin), study design (e.g., qualitative or quantitative, cross‐sectional or longitudinal study), participant characteristics of the dietitian (e.g., the number of participants) and older adults (e.g., age and settings such as RACFs and in home care), methodology (including recruitment, methods, e.g., survey, interview, focus group), and both qualitative and quantitative data exploring dietitians' KSA working in RACFs and home care services. Three researchers (KB, RR and LB) reviewed and discussed discrepancies in the extracted data for consensus.

#### Quality appraisal

The Johanna Briggs Institute Checklist for Qualitative Research^11^ and Checklist for Analytical Cross‐Sectional Studies^12^ were used to assess the methodological quality of the studies and whether any bias may exist within study design, conduct and analysis. The qualitative checklist included 10 questions with *yes*, *no*, *unclear* and *non‐applicable* response options and a final overall appraisal question with responses including *include*, *exclude* and *seek further information*. The quantitative cross‐sectional checklist included eight questions with the same response options as the qualitative response and the same overall appraisal question. The studies were appraised in duplicate (KB and RR) and, if any discrepancies arose, a third researcher (LB) was involved.

#### Data synthesis

Data from studies were analysed thematically using meta‐synthesis. Meta‐synthesis offers a novel finding through integrative interpretation of the results of each study reviewed.^13^ Data were analysed using an iterative comparison of studies to identify reoccurring themes and subthemes.^9^ Findings of the studies were independently read several times, placed into a table, and then compared across the studies to identify relationships, themes and subthemes.^9^

## RESULTS

### Overview

**Figure 1:**
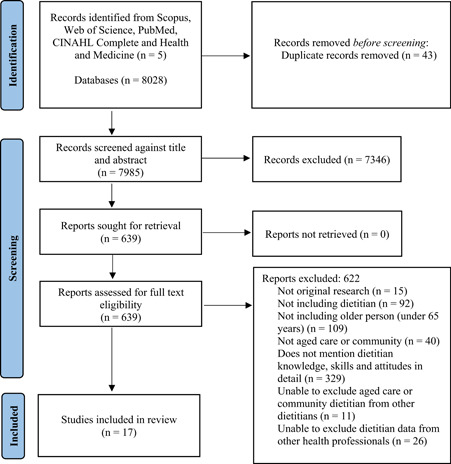
Preferred Reporting Items for Systematic Reviews and Meta‐Analyses flow diagram of included studies

The PRISMA flow diagram is shown in Figure 1. Initially, 7985 studies were screened by title and abstract and, of these, 639 studies were screened using their full‐text version. Seventeen studies (comprising of dietitians, *n* = 2635; student dietitians, *n* = 9) met the inclusion criteria, and study details are reported in Table 1. Studies comprised of descriptive and analytical cross‐sectional designs. Methods included surveys (*n* = 10),^14^, ^15^, ^16^, ^17^, ^18^, ^20^, ^21^, ^22^, ^24^, ^28^ interviews (*n* = 6)^19^, ^25^, ^26^, ^27^, ^29^, ^30^ and focus group (*n* = 1).^23^ Participant numbers ranged from *n* = 1^25^ to *n* = 1281.^15^ Twelve studies included dietitians in RACFs and five studies explored dietitians working within home care services and studies were published between 1986^18^ to 2021.^27^ The location of the studies were conducted in the USA (*n* = 6),^15^, ^18^, ^19^, ^20^, ^22^, ^28^ Canada (*n* = 3),^14^, ^26^, ^29^ Australia (*n* = 2),^16^, ^24^ UK (*n* = 2),^23^, ^25^ Israel (*n* = 2),^17^, ^27^ Japan (*n* = 1)^21^ and the Netherlands (*n* = 1).^30^

**Table 1:** Studies (*n* = 17) exploring dietitians' knowledge and attitudes of working with older adults in RACFs and home care services

First author surname (year) Country	Study design and method	Relevant aim^a^	Focus of study	Participants (sample size), setting	KSA element discussed
*Quantitative* a					
Black et al (2013)^14^ Canada	Cross‐sectional online survey	To explore current practices and workforce related issues amongst LTC dietitians	Challenges for LTC dietitians	Dietitians (*n* = 75) nursing homes	Attitudes
Chao et al (2008)^15^ USA	Cross‐sectional survey	To learn whether registered dietitians working in assisted‐living facilities rate the Food and Nutrition Care Indicators Checklist^b^ as highly important	Opinions on food and nutrition care indicators	Dietitian (*n* = 1281) nursing homes	Attitudes
Craven et al (2016)^16^ Australia	Cross‐sectional online survey	To explore malnutrition screening practices by dietitians working in the community setting	Malnutrition screening	Dietitians (*n* = 133) home	Attitudes
Endevelt et al (2007)^17^ Israel	Cross‐sectional survey	To explore Maccabis' dietitians' attitudes and knowledge regarding elderly nutrition	Elderly nutritional factors	Dietitian (*n* = 95) nursing homes	Attitudes, perceived knowledge
Finley and Simons (1986)^18^ USA	Cross‐sectional survey	To identify and compare perception of the role of the consultant dietitian in the nursing homes in Iowa by consultant dietitians	Role perceptions	Dietitians (*n* = 69) nursing homes	Attitudes
Finn et al (1991)^19^ USA	Cross‐sectional phone interview	To determine the image and activities of consultant dietitians in LTC	Image and role of dietitians	Dietitian (*n* = 328) nursing homes	Attitudes, perceived knowledge
Gilbride and Simko (1986)^20^ USA	Cross‐sectional survey	To describe the functions of a dietitian's role as perceived by dietitians and to examine the level of consensus among dietitians about the importance of each of the role functions	Role functions of dietitians	Dietitians: full time (*n* = 200) part time (*n* = 125) nursing homes	Attitudes
Hirakawa et al (2003)^21^ Japan	Cross‐sectional survey	To understand the current home visit system and identify any barriers to the spread of the system	Home care services	Dietitians (*n* = 129) home	Attitudes and perceived knowledge.
Welch et al (1988)^22^ USA	Cross‐sectional survey	To describe the amount and adequacy of time spent by consultant dietitians in role functions and to identify foodservice and nutrition care problems in nursing homes	Time in role functions and foodservices and nutrition care problems	Dietitians (*n* = 43) nursing homes	Attitudes
*Qualitative*					
Avgerinou et al (2020)^23^ UK	Cross‐sectional focus group	To explore the views and practices of dietitians on the management of malnutrition in frail older people	Malnutrition management	Dietitian (*n* = 2) home	Attitudes
Beelen (2017)^24^ Netherlands	Cross‐sectional interviews	To better understand how protein‐enriched products would fit in the current treatment of undernutrition	Treatment of undernutrition	Dietitians (*n* = 3) nursing homes	Attitudes
Craven et al (2017)^24^ Australia	Cross‐sectional survey	To identify dietitians' barriers and enablers to malnutrition screening of community living older adults	Malnutrition screening	Dietitians (*n* = 92) home	Attitudes
Mole et al (2019)^25^ UK	Cross‐sectional interview	To understand the experiences and perspectives relating to nutritional care of health care professionals and home care workers, who interact with people living with dementia at home	Nutritional care for people living with dementia	Dietitian (*n* = 1) home	Attitudes
Osinga and Keller (2013)^26^ Canada	Cross‐sectional interview	To examine dietetic students' experiences who volunteered or were paid meal helpers in Ontario LTC homes	Experiences providing meal assistance	Student dietitians (*n* = 9) nursing homes	Attitudes
Sheffer‐Hilel et al (2021)^27^ Israel	Cross‐sectional interview	To explore dietitians' experience of nutrition‐focused physical examination‐oriented^c^ change and its implementation into their routine practice	Change to practice (about new nutrition‐focused physical examination)	Dietitians (*n* = 18) nursing homes	Attitudes, perceived knowledge
Suarez and Curry (1989)^28^ USA	Cross‐sectional survey	To understand role functions, attitudes and perceptions of dietitians working in LTC homes	Role functions of dietitians	Dietitians (*n* = 30) nursing homes	Attitudes
Wassink and Chapman (2010)^29^ Canada	Cross‐sectional interview	To gain insight into LTC dietitians' experience of clinical practice	Perspectives of their role and identifying success‐related factors	Dietitians (*n* = 11) nursing homes	Attitudes

Abbreviations: KSA, knowledge, skills and attitudes; LTC, long‐term care; RACFs, residential aged care facilities.

^a^ Relevant aim: aims were adapted from included studies to provide overview of data extracted.

^b^ Food and Nutrition Care Indicators Checklist: Food and Nutrition Care Indicators Checklist indicator for evaluating food and nutrition services.

^c^ Nutrition‐focused physicals examination: the assessment of patients' nutritional status based on a physical examination of oral health.

All studies all explored dietitians' attitudes towards their role, three studies examined perceived knowledge, although no study objectively measured dietitians' skill levels. Knowledge and attitudes were investigated in relation to the role of dietitians (*n* = 7),^15^, ^18^, ^19^, ^20^, ^22^, ^28^, ^29^ malnutrition/undernutrition screening or management (*n* = 4),^16^, ^23^, ^24^, ^30^ challenges or changes to practice (*n* = 2),^14^, ^27^ factors affecting the nutrition status of older adults (*n* = 2),^17^, ^25^ student dietitians' experience providing meal assistance (*n* = 1)^26^ and conduct of home visit services (*n* = 1).^21^

The methodological quality of studies is reported in Table 2. Quantitative studies scored ‘yes’ to 2/8^21^ to 6/8^20^, ^24^ criteria; qualitative studies scored ‘yes’ to 2/10^22^ to 10/10^26^ criteria. Quality ratings for quantitative studies were reduced primarily as a result of not clearly defining the inclusion criteria, the exposure not measured validly and reliably, and confounding factors were not identified. Quality rating deductions for qualitative studies included a lack of congruity with the philosophical perspective and research methodology, no statement addressing any research influence on the research or locating the researcher culturally or theoretically. All studies were included irrespective of methodological quality checklist scores as a result of the paucity of literature available.

**Table 2:** JBI critical appraisal checklist for analytical cross‐sectional studies (*n* = 9) and qualitative research (*n* = 8)

First author (year)	Q1	Q2	Q3	Q4	Q5	Q6	Q7	Q8	Q9	Q10	Total^a^	Overall appraisal include
*Quantitative* b												
Black et al (2013)^14^	Y	Y	N/A	Y	N/A	N/A	Y	Y	–	–	5	✓
Chao et al (2008)^15^	Y	N	N/A	Y	N	N/A	Y	Y	–	–	4	✓
Craven et al (2016)^16^	Y	Y	N/A	Y	Y	N	Y	Y	–	–	6	✓
Endevelt et al (2007)^17^	N	Y	N/A	U	Y	Y	Y	Y	–	–	5	✓
Finley and Simons (1986)^18^	Y	Y	N/A	Y	N	N/A	Y	Y	–	–	5	✓
Finn et al (1991)^19^	Y	N	N/A	U	N	N/A	Y	Y	–	–	3	✓
Gilbride and Simko (1986)^20^	Y	Y	N/A	Y	Y	N	Y	Y	–	–	6	✓
Hirakawa et al (2003)^21^	N	Y	N/A	Y	N	N/A	U	N/A	–	–	2	✓
Welch et al (1988)^22^	N	Y	N/A	Y	Y	N	Y	Y	–	–	5	✓
*Qualitative* c												
Avgerinou et al (2020)^23^	N	Y	Y	Y	Y	Y	N	Y	Y	Y	8	✓
Beelen (2017)	N	Y	Y	Y	Y	Y	N	U	Y	Y	7	✓
Craven et al (2017)^24^	N	Y	Y	Y	Y	N	N	Y	Y	Y	7	✓
Mole et al (2019)^25^	Y	Y	Y	Y	Y	Y	Y	Y	Y	Y	10	✓
Osinga and Keller (2013)^26^	Y	Y	Y	Y	Y	N	U	Y	Y	Y	8	✓
Sheffer‐Hilel et al (2021)^27^	Y	Y	Y	Y	Y	N	N	Y	Y	Y	8	✓
Suarez and Curry (1989)^28^	N	U	U	U	U	N	N	Y	N	Y	2	✓
Wassink and Chapman (2010)^29^	Y	Y	Y	Y	Y	U	N	Y	Y	Y	8	✓

Abbreviations: JBI, Johanna Briggs Institute; N, no; N/A, not applicable; Q, question; U, unclear; Y, yes.

^a^ Total number of ‘Yes’ responses.

^b^ JBI Critical Appraisal Checklist for Analytical Cross‐Sectional Studies — Q1: Were the criteria for inclusion in the sample clearly defined? Q2: Were the study subjects and the setting described in detail? Q3: Was the exposure measured in a valid and reliable way? Q4: Were objective, standard criteria used for measurement of the condition? Q5: Were confounding factors identified? Q6: Were strategies to deal with confounding factors stated? Q7: Were the outcomes measured in a valid and reliable way? Q8: Was appropriate statistical analysis used?

^c^ JBI Critical Appraisal Checklist for Qualitative Research — Q1: Is there congruity between the stated philosophical perspective and the research methodology? Q2: Is there congruity between the research methodology and the research question or objectives? Q3: Is there congruity between the research methodology and the methods used to collect data? Q4: Is there congruity between the research methodology and the representation and analysis of data? Q5: Is there congruity between the research methodology and the interpretation of results? Q6: Is there a statement locating the researcher culturally or theoretically? Q7: Is the influence of the researcher on the research, and vice versa, addressed? Q8: Are participants, and their voices, adequately represented? Q9: Is the research ethical according to current criteria or, for recent studies, and is there evidence of ethical approval by an appropriate body? Q10: Do the conclusions drawn in the research report flow from the analysis, or interpretation, of the data?

### Meta‐synthesis

Five themes were developed inductively: (1) recognising their contribution as dietitians; (2) lacking clarity about the boundaries of their role; (3) all team members have a role to play in nutrition care; (4) assumptions and biases about working with older people; and (5) needing to build capacity in the workforce.

#### Recognising their contribution as dietitians

The first theme highlighted dietitians' strong sense of value in contributing to RACFs and home care services. Dietitians reported taking their role in aged care ‘seriously’^26^, ^29^ and recognised the distinct value of their role within clinical care teams.^26^, ^29^ In one study where student dietitians provided practical feeding support to residents in RACFs, students expressed they undertook their activities to a high standard in the pursuit of providing autonomy and dignity to the older adults they were caring for.^26^ Student dietitians valued the opportunity to gain experience in the sector, not just for future employability but because of the apparent positive impact their actions had on fellow staff and patients.^26^

#### Lacking clarity about the boundaries of their role

The second theme emphasised dietitians' perceived lack of role clarity working in RACFs and home care services. Dietitians described their roles and tasks as variable, everchanging and multi‐faceted, to the extent that their role may be unclear to others.^29^ For example, one dietitian recalled that their facility manager once asked, ‘*Can I get a list of what (services) you provide?*’.^29^ Other dietitians explained their tasks as mostly ‘self‐appointed’ or ‘self‐directed’, with one dietitian reporting, ‘*I always make my job … there has never been a job description for me and … I have never been trained for a job, ever*’.^29^ Several other studies supported the notion of lack of clarity for dietetic roles, as each study described different role functions, including providing nutrition care, working with food service, completing in‐service education for staff and maintaining and conducting quality assurance audits.^18^, ^19^, ^20^, ^27^, ^28^, ^29^ Dietitians also perceived a difference between actual and desired tasks, specifically that spending time with patients was a high priority but not always possible because of their other duties.^18^, ^28^ Dietitians' lack of role clarity contributed to concerns about how other team members perceive their role. One dietitian expressed ‘*I have so many areas of responsibility that I am not good at anything … it's very frustrating*’.^27^ These concerning attitudes have persisted over time, with one survey in the year 1989 finding that most (*n* = 22; 73%) participants were frustrated in their role, some (*n* = 9; 30%) felt depressed about their role, and half (*n* = 15; 50%) were satisfied in their role.^28^ More recently, dietitians rated other professions higher than their own regarding their position being viewed as ‘necessary/vital/important’ and ‘helpful and cooperative’.^19^

#### All team members have a role to play in nutrition care

The third theme focuses on dietitians' view that *all* staff in RACFs and home care services can influence the nutrition status of older adults and therefore have a role to play in nutrition care. Dietitians acknowledged that it is not just the role of dietitians to deliver nutrition care and implement nutrition plans or interventions.^17^, ^25^ One study described nutrition as a ‘*team effort*’ and further emphasised that ‘*nutritionists are totally ineffective without nursing behind them*’, stemming from the lower workforce size and subsequent capacity of dietitians.^29^ Dietitians recognised the importance of teamwork; however, they did not feel that all team members supported the role of dietitians in providing nutrition care. For example, dietitians perceived that referrals were not always timely^30^ or requested.^21^ In a survey study, dietitians reported that team members did not feel they had sufficient time to contribute to nutrition care,^28^ which would burden their work.^24^ In addition, dietitians faced challenges to the timely provision of nutrition care, including lack of anthropometric or biomedical data^22^ and other staff feeling ‘*undertrained*’ in nutrition considerations.^28^ Dietitians also expressed they were not respected and were not well utilised to meet patient needs.^28^ In the focus groups conducted by Avgerinou *et al*.,^23^ dietitians expressed needing to overcome misinformation that patients received from other team members, which was sometimes perceived as inconsistent and non‐evidence‐based. As such, dietitians highlighted the potential positive impact of additional education and training on nutrition for all health professionals and team members, which would support dietitians in carrying out their role to a high standard.^22^, ^24^

#### Assumptions and biases about working with older people

The fourth theme highlighted dietitians' assumptions and biases about working with older people. In a survey of 1281 dietitians, only half of the dietitians (53%) perceived older people ‘*would be able to make wise dietary choices on their own but that they needed education and counselling*’.^15^ Very few (31%) thought older adults could ‘… *make dietary choices on their own without assistance*’, and some (16%) thought that older adults were ‘*unable to make dietary choices on their own*’.^15^ A similar finding occurred through a survey from Israel, with almost half (49.5%) of the dietitians strongly disagreeing that older people ‘*can't change their nutritional habits*’, whereas 24% slightly agreed that older people could not change their nutritional habits.^17^ Furthermore, a cross‐sectional survey, reported differences in how dietitians described older people, with some dietitians stating they're ‘*lovable and fun*’, yet others defined them as ‘*irritable, pitiable, and confused*’.^28^ These papers suggest that dietitians have assumptions and biases about older people, including their ability to make positive changes to their diet, the amount of nutrition education and counselling they require, and their overall demeanour.

#### Needing to build capacity in the workforce

The fifth theme highlighted a clear need to increase RACFs and in‐home care services' workforce capacity because dietitians reported a lack of time, funding and professional development opportunities. Dietitians reported insufficient time to complete required tasks.^14^ Concern over workforce capacity has persisted over time, with a cross‐sectional study in 1988 suggesting that 74% of dietitians in their study had insufficient time spent within the nursing home to fulfil professional responsibilities.^22^ Dietitians also mentioned workload difficulties because of a lack of time, human resources and increased workload.^27^ In five studies, dietitians suggested that more job opportunities are needed in RACFs and home care services.^16^, ^19^, ^21^, ^25^, ^27^ A dietitian also mentioned a shortage of employment positions for dietitians in an interview conducted in Israel; ‘*Unfortunately … the current standardisation (hours and workload requirements) doesn't allow me to work to the extent and quality I would like*’.^27^ Furthermore, dietitians perceived that the organisations or businesses that commission the services do not allocate sufficient paid time to monitor their patients effectively.^19^, ^25^ Dietitians also shared a lack of confidence as some reported fewer positive responses about their knowledge in general compared to other health professionals.^19^

Dietitians reported they need to build knowledge with new tasks and highlight that they need time to develop professionally.^27^ Despite a perceived lack of knowledge around new tasks and poorer ratings of perceived knowledge, 63% of dietitians rated their overall perceived knowledge about older adult nutrition as ‘good’.^17^ Furthermore, dietitians revealed that they lack professional development opportunities to improve this knowledge.^27^ The barriers to knowledge development described by dietitians can negatively impact on dietitian‐patient relationships and the dietitians' ability to improve the patient's quality of life.^27^

## DISCUSSION

This integrative review synthesised a modest body of literature on dietitians' knowledge and attitudes regarding working with older adults in RACFs and home care services. Collectively, this review contributes to the small amount of literature about dietitians working with older adults in RACFs and their homes. Dietitians and student dietitians recognised their contributions to the clinical care team and took their role seriously in RACFs. The review did not include any studies with nutritionists or explore any aspect of dietitians or student dietitian skills as a result of an absence of literature.

Dietitians reported challenges understanding the boundaries of their role working with older adults. Lack of role clarity is consistent in a recent study by Hickson et al.^31^ where dietitians revealed desire for visibility, role clarity and future growth. Dietitians Australia released a role statement for RACF dietitians in July 2021 to provide consensus and clarity.^32^ This role statement provides dietitians and other health professionals with a comprehensive list of knowledge and skills expected of dietitians working in RACFs.^32^ The dissemination and implementation of the role statement have not been formally evaluated and should be considered in the future. Additionally, a role statement for dietitians working in‐home care services may provide similar clarity to dietitians and other staff members. Enhanced role clarity by integrating interprofessional education and clarity throughout organisations is imperative to reduce staff burnout, increase retention, and ensure the delivery of quality and safe care.^33^

Dietitians heralded that all team members have a role in nutrition care. However, limited involvement of staff in nutrition care in RACFs has been reported in a cross‐sectional survey study by Beattie et al.^34^ This survey revealed that 83% of nursing, personal care, catering and therapy RACF staff considered nutrition care and assessment important despite lacking nutrition knowledge (with a mean score of 47%) and only 53% reported carrying out nutrition activities.^34^ Furthermore, a 2020 systematic review on malnutrition practices in older adults revealed that other health professionals' time for, and knowledge of, screening policies is inadequate and negatively affects the impact of dietitians.^35^ Cave et al.^36^ highlighted that teamwork between all staff is required to encourage adequate dietary intake in older adults. The lack of nutrition knowledge and prioritising nutrition care was also a key finding of the 2021 Royal Commission into Quality and Safety in Aged Care in Australia.^2^ As such, the findings of this integrative review support Dietitians Australia recommendation to review and update certificate courses for RACF care staff to include a component of nutrition to their education to enhance knowledge.^37^ Including nutrition in these courses could increase knowledge and skills relating to nutrition care for older adults,^37^ thus enhancing the nutrition status and health outcomes of this important population.

Dietitians appear to have assumptions and biases regarding older people. Assumptions and biases of older people and aging are not uncommon amongst health care professionals and have been well documented in the literature.^38^, ^39^ Stereotypes go beyond the older person and can also relate to the setting. Lordly and Taper^40^ mixed‐methods survey study revealed that dietitians have negative attitudes towards being trained and/or working in long‐term care. It was revealed that long‐term care dietitians feel ‘lesser’ than acute care dietitians.^40^ Negative attitudes about working in these settings are consistent with two survey studies revealing low work preferences for student dietitians working with older people.^41^, ^42^ Students reported concerns and fears about working with older people.^41^

To minimise these negative assumptions and biases, interventions targeting the student experience and knowledge need to be developed. For example, increasing exposure through placements, volunteering, work experience or a guided experiential assignment^43^ and providing adequate undergraduate theory on nutrition for older people could reduce these assumptions and biases in future dietitians. Strengthening the RACF and home care workforce for dietitians may assist in optimising attitudes and encouraging more dietitians to work in these settings. Healthcare systems need to continue to work toward patient centred anti‐ageism care and ensure all older people have access to care,^38^ including dietitian services. A key area to provide education and initiative to reduce ageism is through universities where future health professionals are trained.^38^

Dietitians highlighted the need for building capacity through more funding, time and professional development opportunities for dietitians working in RACFs and home care services. The identified workforce capacity‐building areas are consistent with the recent 2021 Royal Commission into Quality and Safety in Aged Care in Australia findings of allied health professionals.^2^ Based on the Royal Commission recommendations, Dietitians Australia also advocates for dietitians to see all residents for 1 h per resident, each month in RACFs and increase older persons' access to dietitians' within home care settings.^8^ A 2016 survey of 150 long‐term care dietitians in Ontario, Canada revealed that 89% of dietitians could not complete all required responsibilities within the mandated Registered Dietitian staffing time of 30 min per resident per month.^44^ By increasing time at RACFs to a recommended 45 min per resident per month, dietitians reported that it would allow for more timely follow‐ups on clinical issues, communication with residents and families, interprofessional communication, staff education, quality improvement activities and other proactive roles.^44^ Despite this survey's results encouraging the increase in mandated hours, the mandated ratio in Ontario has remained unchanged. Additional time could allow for more opportunities to build capacity in the workforce. As such, future research needs to examine the impact of additional dietetic time on older adults' dietary intake, health outcomes and economic evaluation of this service. Moreover, continuing to advocate for dietitians to be employed full‐time in RACFs and patients having more access to home care dietitians will allow for ongoing support from the dietitian and the opportunity for in‐service training for all staff.

Dietitians' actual knowledge and skills were not synthesised from this review because they were not objectively measured in the original research studies. Although one study provided insight into the perceived knowledge of dietitians, which is consistent with previous surveys exploring actual knowledge,^41^, ^42^ it is important to recognise that actual and perceived knowledge may not always be congruent.^45^ Perceived knowledge is an individual's assessment of the knowing information; as such, individuals may overestimate, underestimate or accurately rate their perceived knowledge following actual knowledge. In addition, perceived knowledge often depends on individual variables (such as self‐esteem and self‐monitoring) and contextual variables (such as topic area and nature of information). Therefore, it is an important consideration when accessing actual knowledge. Thus, a dietitian's knowledge (both perceived and actual) and skills about working in RACFs and home care settings is required to understand the knowledge and skills gap to target further education and training to current and upcoming dietitians. Further research is needed to understand dietitians' knowledge and skills of dietitians working in RACFs and home care settings to ensure dietitians provide optimal nutrition care to all patients.

There are notable strengths and limitations of the review. A strength of this review is the variety of designs in 17 studies. The range of methodological designs and objectives provides a broad overview of dietitians' attitudes and some insight into perceived knowledge working in RACFs and home care. The quality of this review has been maximised at all stages of the review by using systematic and rigorous processes. The processes undertaken include two independent researchers screening title and abstract and full text, cross‐checking all elements of data extraction, and a third reviewer's availability to assist if any discrepancies arise. The methodological process used was to reduce any bias and potential complexities associated with the synthesis of qualitative and quantitative findings. The strength of using the iterative process of meta‐synthesis allowed identification of emerging themes, which were reviewed and revised by all of the investigators. However, the methodological quality, language of study and date of publications of studies were not a criterion for inclusion or exclusion. Thus, the results of this review must be interpreted with caution as it includes lower‐quality studies, a translated article and older studies. Additionally, the findings were often reported in context to a particular component or area of RACFs and home care services, such as malnutrition management or role functions of dietitians. Explicating examining dietitians' attitudes working in these settings may elicit different results.

## CONCLUSIONS

Dietitians recognised the important contribution of their role to caring for older adults; however, they experience low clarity about the boundaries of their role. Dietitians appear to have positive and negative assumptions and biases about older people. Moreover, dietitians have recognised issues in the workforce, with not all team members prioritising nutrition care despite dietitians recognising all members have a role. Dietitians recognised that the dietetic workforce needs to build capacity. Additional research is needed to understand dietitians' knowledge and skills regarding working in RACFs and home care services to provide insight into future research and initiatives. Future directions could include evaluating dietitians' role in aged care, reviewing education and training and practical opportunities for student dietitians, and assessing the impact mandated dietitian hours have on an older person's dietary intake and nutrition.

## CONFLICTS OF INTEREST

Lauren Ball is on the editorial board for the *Journal of Human Nutrition and Dietetics*.

## AUTHOR CONTRIBUTIONS

Karly Bartrim contributed to the conception and design of the research, the collection and analysis of the data, and created the original draft and reviewed and edited subsequent versions. All authors contributed to the design of the research, to the analysis and interpretation of the data, and to writing, reviewing and editing. All authors approved the final version of the manuscript submitted for publication. All authors declare that the content of the manuscript has not been published elsewhere.

## TRANSPARENCY DECLARATION

The lead author affirms that this manuscript is an honest, accurate and transparent account of the study being reported. The reporting of this work is compliant with PRISMA guidelines. The lead author affirms that no important aspects of the study have been omitted and that any discrepancies from the study as planned have been explained. The review was not registered.
